# Fracture Resistance and Failure Mode of Mandibular Molar Restored by Occlusal Veneer: Effect of Material Type and Dental Bonding Surface

**DOI:** 10.3390/ma14216476

**Published:** 2021-10-28

**Authors:** Walid Al-Zordk, Alaa Saudi, Abdelraheem Abdelkader, Mansoura Taher, Mohamed Ghazy

**Affiliations:** 1 Fixed Prosthodontics Department, Faculty of Dentistry, Mansoura University, Mansoura 35516, Egypt; alaasaudi98@gmail.com (A.S.); abdelrheem_ali@yahoo.com (A.A.); mghazy@mans.edu.eg (M.G.); 2 Fixed Prosthodontics Department, Faculty of Dentistry, Horus University, Damietta 34511, Egypt; 3 Conservative Dentistry Department, Faculty of Dentistry, Mansoura University, Mansoura 35516, Egypt; taher_mans@yahoo.com

**Keywords:** bonded restoration, tooth wear, table-top, non-retentive, resin cement

## Abstract

This study assesses the effect of the material type (lithium disilicate, zirconia, and polymer-infiltrated ceramic) and dental bonding substrates (dentin, dentin with intra-coronal cavity, and dentin with composite filling) on the fracture resistance and failure mode of molars restored by occlusal veneers. Methods: Ninety occlusal veneers, fabricated from either lithium disilicate, zirconia, or polymer-infiltrated ceramic, were adhesively bonded to teeth prepared with either dentin, dentin with intra-coronal cavity, or dentin with composite filling. All specimens were thermally aged (5000 cycles), then load cycled (120,000 cycles). Each specimen was subjected to a compressive load through fracture, then was examined (×20) to identify the fracture type. Data were statistically analyzed. Results: Material type and dental substrate had no significant effect on the fracture resistance of adhesively retained occlusal veneer restorations. For each material, no significant differences were found between veneers bonded to dentin, dentin with intra-coronal cavity, and dentin with composite filling. Additionally, within each bonding substrate, there were no significant differences between lithium disilicate, zirconia, and polymer-infiltrated ceramic veneers. The adhesive failure was recorded mainly with zirconia occlusal veneer restorations. Conclusions: Considering the fracture results, lithium disilicate, zirconia, and polymer-infiltrated ceramic occlusal veneers perform well whatever the type of dental bonding surface. When the dental bonding surface varies, different occlusal veneer materials should be considered. Occlusal veneers bonded to dentin, dentin with composite filling, or dentin with an intra-coronal cavity exhibited a fracture resistance exceeding the average human masticatory forces in the molar area.

## 1. Introduction

Occlusal defects could be induced by the destruction of occlusal enamel and exposure of the underlying dentin as a result of pathological and functional problems such as tooth wear or caries [1]. Loss of occlusal contacts can also be caused by problems such as an open occlusal relationship with or without orthodontic treatment [2]. The management of such lesions presents a challenge and could be treated with the aid of inlay, onlay or crown restorations [3,4]. Unfortunately, such treatment methods can be very destructive [4]. As adhesive bonding procedures improve, minimally invasive approaches are preferred because they provide many benefits, including optimum preservation of sound tooth structure, preservation of pulp vitality, and reduction of post-operative sensitivity [5,6,7,8,9]. Occlusal veneers have gained more popularity as a treatment modality for severely worn teeth [10,11]. With defects in the occlusal surface, the degree of damage to worn teeth is irregular [12]. The preparation configurations of the occlusal veneer restorations are adjusted to repair those defects [13,14,15,16,17].

The combination of improved dental adhesive technologies and restorative dental materials with enhanced mechanical strength for use of thin thicknesses allows for the minimally invasive replacement of lost hard tooth structures [18,19,20]. Among monolithic ceramic restorations, the clinical effectiveness of lithium disilicate restorations has been documented and it has been advocated for the rehabilitating teeth with minimal or no preparation to restore erosive or abrasive lesions where replacement of damaged enamel is indicated [21]. Zirconium oxide ceramics have superior mechanical properties and have shown promising results as fixed restorations [22,23,24,25,26,27]. Polymer-infiltrated ceramic materials are less brittle than ceramics, and it has been assumed that they have good resistance to dynamic fatigue. However, wear, discoloration, and reduced fracture strength are concerns [28,29,30].

When bonded to the tooth, the restorative materials should be strong enough to resist occlusal forces [22]. Ceramic fractures have been identified as a major cause of restoration failure [29]. Several factors affect the fracture resistance of ceramic restorations, including the type and thickness of ceramic material, luting technique, and occlusal loads [22,31]. Another critical aspect that can affect the fracture resistance of a ceramic restoration is the tooth preparation [32,33]. In addition, the fracture resistance of a ceramic restoration is influenced by the quality of bonding at the restoration-tooth interface [34,35,36]. Furthermore, the type of bonding surfaces and the ability to achieve durable bonding are important factors in the restoration’s success [37]. However, teeth that require occlusal veneers seldom have only one type of hard tooth substance [38]. With occlusal veneers, the dental bonding surfaces can vary greatly, and may be purely enamel, dentin, or composite fillings, and each of these bonding substrates could affect the fracture resistance differently [34,38,39]. The strength of tooth-restoration complex is influenced by the elastic moduli of the material and substrate, as well as the material’s flexural resistance [40,41].

The fracture resistance testing is crucial for determining how teeth react to high-intensity loading [11,15,42,43,44,45,46]. The aim of this research was to study how occlusal veneer materials (lithium disilicate, zirconia, and polymer-infiltrated ceramic) and dental bonding substrates (dentin, dentin with intra-coronal cavity, and dentin with composite filling) affected the fracture resistance and failure mode of mandibular molars restored with occlusal veneers. The first null hypothesis was that the fracturing resistance of occlusal veneers bonded to mandibular molars would not be affected by various restorative materials. The second null hypothesis was that the dental bonding surface would not impair fracture resistance.

## 2. Materials and Methods

Following approval from the local Research Ethics Committee (No. A19110220), ninety mandibular first molars, extracted for periodontal purposes, were obtained for this experiment. The collected teeth were cleaned from any superficial stains and calculus using an ultrasonic scalar (UDS-K, Guilin Woodpecker Medical Instrument Co. Ltd., Guangxi, China). Under proper lightening, the teeth were examined under magnification (×20) to detect any cracks or fractures. The selected teeth were free from cracks, caries, old restorations, and other defects. The teeth were cleaned and stored in a standardized saline solution (Sodium Chloride BP 0.9%, Fibco, Alexandria, Egypt) at room temperature until use. The materials used in the current study are presented in Table 1.

Study grouping: The teeth were randomly divided into nine groups (*n* = 10) based on material type (lithium disilicate, zirconia, and polymer-infiltrated ceramic) and dental bonding surface (dentin, dentin with intra-coronal cavity, and dentin with composite filling). Table 2 shows the study grouping.

**Figure 1:**
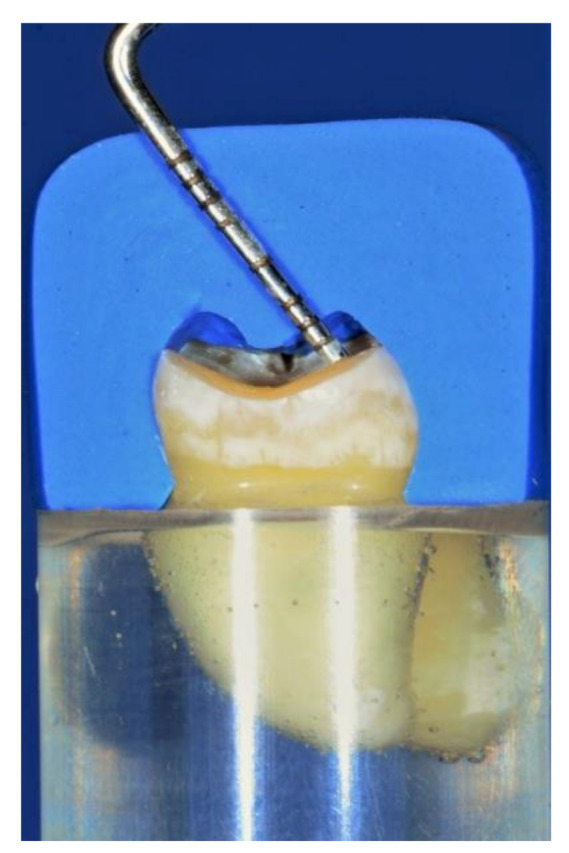
The pre-preparation silicon index was used to check the amount of tooth reduction (1 mm to simulate the wear and 1 mm to create a space for a 1 mm thickness restoration).

Teeth preparation: Each tooth was positioned vertically in an auto-polymerizing resin material (Kemapoxy 150, CMB, Cairo, Egypt), with the cemento-enamel junction 2 mm above the resin surface. Then, using additional silicone impression material (Presigum, President Dental GmbH, Allershausen, Germany), a pre-preparation silicon index was fabricated for each tooth. The tooth preparation was performed using a straight hand-piece (Traus AT-II, Saeshin Precision Co., Daegu, Korea) with the aid of a paralleling device (Dentalfarm A3006 B, Turin, Italy). All preparations were performed by a single operator (A.S). For all teeth, the occlusal surface was reduced by 2 mm (1 mm to simulate the occlusal wear and additional 1 mm to create a space for the restoration) at the cusp tip and central groove, following the occlusal anatomy with 150° divergence angle between cusps as shown in Figure 1 [11,34]. For LC, ZC, and PC groups, the intra-coronal cavities were prepared using a diamond stone (856 blue, Intesiv SA, Montagnola, Switzerland). The intra-coronal cavity had 1 mm pulpal depth, 2 mm bucco-lingual width, 1.6 mm away from the proximal marginal ridge, and an 8-degree wall taper. For LF, ZF, and PF groups, the intra-coronal cavities were also prepared as previously described, then a universal adhesive (Tertric N-Bond Universal, Ivoclar Vivadent, Schaan, Liechtenstein) was applied over each intra-coronal cavity and light-activated (Coltolux LED, Coltene/Whaledent Inc., Cuyahoga Falls, OH, USA) at each surface for 20 s following the manufacture’s recommendations. Then, the intra-coronal cavities (LF, ZF, and PF groups) were restored using a composite resin (Tetric N-Ceram Bulk-Fill, Ivoclar Vivadent, Schaan, Liechtenstein) and light activated for 20 s. Finally, all prepared teeth were smoothened and finished using a tapered round-end diamond stone (856 yellow, Intesiv SA, Montagnola, Switzerland).

Fabrication of occlusal veneers: Each tooth was scanned using an extra-oral scanner (ldentica Hybrid, Medit Corp., Seoul, Korea). Each veneer restoration was designed using CAD-CAM software (Valletta 2015 v2.2, Exocad GmbH, Darmstadt, Germany). The cement gap was set at 50 μm. The thickness of all veneer restorations was 1 mm (except LC, ZC, and PC groups in cavity projection areas). Then, the restorations were milled (Coritec 250i, Imes-Icore GmbH, Eiterfeld, Germany) from lithium disilicate (IPS e.max CAD, Ivoclar Vivadent, Schaan, Liechtenstein), zirconia (Katana UTML Kuraray Noritake Dental Inc., Tokyo, Japan), and polymer-infiltrated ceramic (Vita Enamic, VITA Zahnfabrik, Bad Säckingen, Germany).

**Figure 2:**
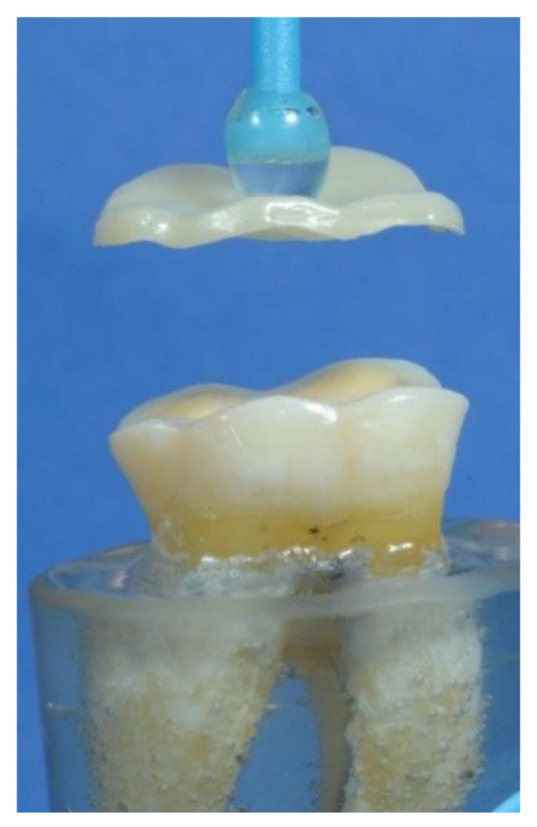
The occlusal veneer during cementation to its corresponding tooth.

Cementation: For lithium disilicate and polymer-infiltrated ceramic occlusal veneers, the intaglio surfaces were treated with hydrofluoric acid (Porcelain Etchant, Bisco inc., Schaumburg, IL, USA) for 20 s. Then, silane (porcelain primer, Bisco inc., Schaumburg, IL, USA) was applied based on the manufacturer’s instructions. For zirconia occlusal veneers, the intaglio surfaces were air-borne particles abraded using a sandblasting unit Renfert Basic eco (Renfert GmbH, Hilzingen, Germany) with 50 µm alumina (Renfert GmbH, Hilzingen, Germany) at a pressure of 2 bars from 10 mm distance and perpendicular to the surface. Then, the primer Z-Primer Plus (Bisco Inc., Schaumburg, IL, USA) was applied according to the recommendations of the manufacturer. For dental substrates, the peripheral enamel of each tooth was selectively acid etched with phosphoric acid (N-Etch, Ivoclar Vivadent, schaan, Liechtenstein). Then, the bonding agent (All-Bond Universal, Bisco Inc., Schaumburg, IL, USA) was applied and cured, according to the manufacturer’s instructions, using a light-emitting diode curing unit (Coltolux LED, Coltene/Whaledent Inc., Cuyahoga Falls, OH, USA) with a mean light intensity of 1000 mW/cm^2^. Before each use of the curing unit, the light intensity was measured using a radiometer (Bluephase Meter II, Ivoclar Vivadent, Schaan, Liechtenstein). Finally, cement (Duo-Link Universal, Bisco Inc., Schaumburg, IL, USA) was added to the fitting surface of the restoration, and the restoration was seated on its corresponding tooth (Figure 2) and placed under a steady load of 10 N [39]. Each surface of the tooth was exposed to light curing (Coltolux LED, Coltene/Whaledent Inc., Cuyahoga Falls, OH, USA) for 40 s. For two weeks, all specimens were kept in water at 37 °C [47].

Artificial aging: All specimens were thermo-cycled for 5000 cycles in a thermo-cycler machine (Thermo-cycler THE-1100, SD Mechatronics, Westerham, Germany) between 5 and 55 °C with 20 s dwell time and transfer time for 10 s [48]. Finally, all specimens were further subjected to dynamic load aging (Robota ACH-09075 DC-T, AD-TECH Technology Ltd., Germany) for 120,000 cycles via a 5.4 mm steel piston at a descending speed of 40 mm/s and 1.6 Hz [49]. After aging, each specimen was examined under magnification (Ergovision 4.0, Ergovision loupes, China) to detect any damage.

**Figure 3:**
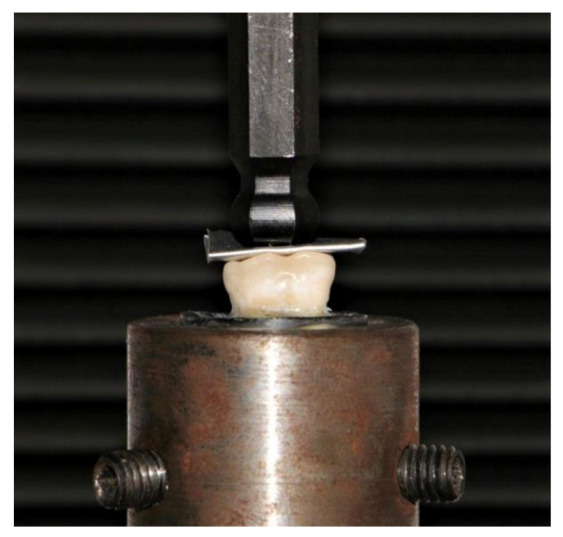
The specimen fixed in the lower compartment of the universal testing machine with the tin foil positioned between the specimen and the metal rod during the fracture test.

Fracture resistance test: The specimen was placed on a universal testing machine (Instron Universal testing machine Model 3345, Canton, MA, USA) and a 0.5 mm tin foil was positioned over the occlusal surface of the restoration to ensure adequate force distribution as shown in Figure 3. Via a 5 mm metallic rod, a compressive load (cross-head speed of 1 mm/mm) was introduced to the occlusal surface of the restoration. The amount of force required to fracture was recorded in Newton (N). To determine the failure mode of each fractured specimen, it was analyzed under ×20 magnification (Olympus SZ 61, Tokyo, Japan) and categorized as Class I (crack within the restoration), Class II (cohesive fracture within the restoration), Class III (adhesive fracture between the restoration and the tooth), or Class IV (longitudinal fracture of the restoration and tooth) [18,50].

Statistical analysis: Shapiro-Wilk testing revealed that data were in a normal distribution. The analysis was performed using SPSS software (v22.0, IBM Corp, Armonk, NY, USA). The significance of the obtained results was judged at 0.05 level. Two-way ANOVA test was used to detect the interaction effect of restoration type and dental bonding surface. Monte Carlo test was used to illustrate the failure pattern percentage of all test groups.

## 3. Results

The mean and standard deviation of the fracture resistance values of the studied groups are shown in Table 3. Two-way ANOVA test showed that material type had no significant effect (*p* = 0.148) on the fracture resistance of the adhesively retained occlusal veneers restorations whatever the type of the underlying bonding surface (Table 4). Within each veneer material, there was no significant difference between veneers bonded to dentin (F = 0.592, *p* = 0.560), dentin with intra-coronal cavity (F = 2.28, *p* = 0.121), and dentin with composite filling (F = 1.93, *p* = 0.164). Additionally, within each dental bonding substrate, there were no significant differences between lithium disilicate (F = 0.669, *p* = 0.521), zirconia (F = 2.25, *p* = 0.124), and polymer-infiltrated ceramic (F = 2.10, *p* = 0.142) veneers.

**Figure 4:**
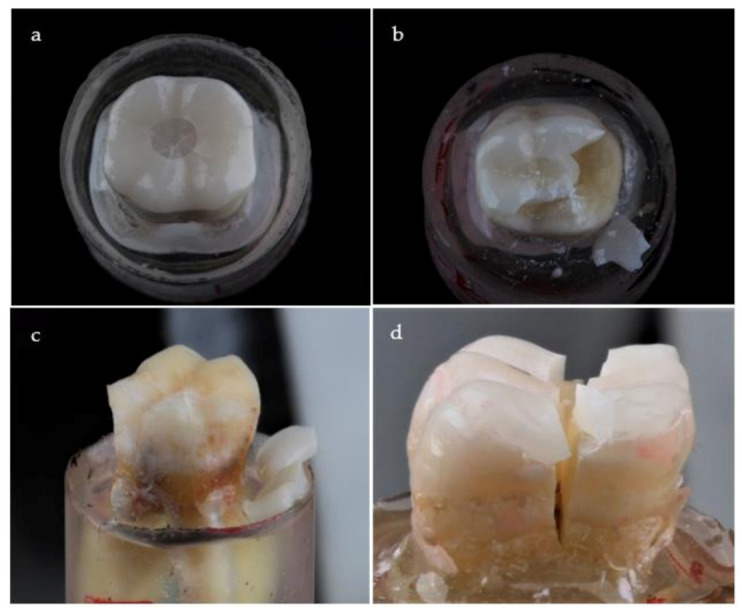
Representative fracture modes of failed specimens: (**a**) Class I: crack formation within restoration without chipping; (**b**) Class II: cohesive fracture within restoration without involving the tooth structure; (**c**) Class III: adhesive fracture between the restoration and tooth; and (**d**) Class IV: longitudinal fracture of the restoration and tooth.

Failure modes among studied groups are presented in Table 5 and Figure 4. The adhesive failure (class III) was recorded only with zirconia occlusal veneers (ZD, ZC, and ZF groups). Monte Carlo test revealed a statistically significant difference (*p* = 0.004) between LD, ZD, and PD groups. The main mode of failure for LD and PD groups was class IV. Additionally, there was a statistically significant difference (*p* = 0.012) between LF, ZF, and PF groups. The main mode of failure for LF, ZF, and PF groups was class IV.

## 4. Discussion

Based on the results of the current study, the hypothesis that the fracture resistance of occlusal veneer is not dependent from its material type was accepted because the material type had no significant effect on the fracture resistance of adhesively retained occlusal veneers. The second hypothesis tested, that the dental bonding surface would not affect the fracture resistance, was accepted.

In the resent study, human natural molar teeth with approximately similar dimensions were selected and standardized tooth reparation was performed. Preparation geometries and tooth morphology have an effect on the fracture resistance and durability of partial-coverage restorations [50]. The occlusal veneer preparations were performed in the dentin to simulate the advanced clinical wear [10]. Although the teeth were reduced by 2 mm to simulate tooth wear and considering the effect of the clinical passive eruption, a ceramic thickness of 1 mm was used in the current study to retain as much tooth structure as possible. The thickness of occlusal veneer restorations should not be less than 0.7 to 1 mm, regardless of the material used [30]. Other studies considered the thickness of 0.8 mm as the appropriate threshold for lithium disilicate occlusal veneers [38,43]. However, it was reported that various occlusal veneer materials should be considered as the amount of available space for restoration varies [11].

Additionally, the occlusal veneer preparation was limited to occlusal surface only without a finish line extended over the axial walls. Occlusal veneer covering only the occlusal surface created minimal stresses within the restoration [14,17]. It was revealed that the existence of a finish line, as well as the form of preparation design in enamel and dentin, had little effect on the fracture resistance of occlusal veneers [34]. Additionally, it was found that stress-bearing areas were generated at the cusp tips under the thin occlusal veneer when prepared with a finish line over the axial walls [11]. In addition, it was found that occlusal veneers which covered just the occlusal surface showed a decrease in maximum stresses in the restoration and higher fracture resistance than traditional full coverage crown and occlusal veneers which partially covered the axial surfaces [14].

In the present study, specimens were subjected to thermal aging (5000 cycles). Thermocycling has been used as a strong method to simulate clinical conditions. Thermal cycling accelerates the diffusion of water by changing the temperature by changing the temperature that produces stress at the interface of two materials. It was reported that the bond strength of universal adhesives to zirconia were reduced after thermocycling [51]. In the current study, a self-adhesive resin cement was used (Duo-Link universal cement system) which is a Bis-GMA-based resin cement and its zirconia primer (Z-prime plus) contains two adhesive monomers (organophosphate monomer and carboxylic acid monomer). It was demonstrated that surface treatment with MDP-containing primer reinforced the bond strength initially and after thermal aging [52]. However, it was reported that the application of priming agents containing hydrophobic phosphate monomer (MDP) resulted in higher post-thermocycling bond strengths than that of priming agents containing carboxylic monomer [53].

The maximum bite force in the posterior area reached 880 N for the normal individuals, and 1120 N for individuals with para-functional [54,55]. In the posterior area, the mean fracture resistance values of the tested occlusal veneer restorations surpassed the human biting forces [56]. As a result, the fracture strength values clearly met the requirements for dental restorations, and reasonable clinical results could be expected from the point of fracture load [38,41].

The fracture resistance of lithium disilicate, zirconia, and polymer-infiltrated ceramic occlusal veneer restorations did not vary significantly, according to the findings of this study. Before cyclic loading, studies investigated various occlusal veneer materials and found no major differences in fracture resistance [18,22]. It was stated that the inherited mechanical characteristics of occlusal veneers did not always equate to the final load potential because the tooth-restoration complex was measured rather than the mechanical properties of the restorative material itself [10]. Since strong adhesive bonding can reinforce weaker ceramics and balance inherent strength differences among various materials, the current findings may be linked to the adhesion properties between the luting cement and the occlusal veneer material [22]. However, a study found that the material type has a substantial impact on the fracture resistance of occlusal veneers [46].

The PD group has the highest fracture resistance value as compared to the LD and ZD groups. This may be due to the fact that polymer-infiltrated ceramics and dentin have a similar modulus of elasticity [28]. However, a study showed a lower fracture resistance value for polymer-infiltrated ceramic occlusal veneers compared to lithium disilicate [18]. Their findings could be related to the different tooth type and the use of self-etch primer [5,9,36]. In addition, a high fracture resistance value was reported for lithium disilicate occlusal veneers, which could be attributed to the non-standardized protocol for cementation and difference in the thickness of the occlusal veneer [42,44,45].

In the current study, lithium disilicate occlusal veneer bonded to dentin showed a high fracture value. Valenzuela et al. [41] reported similar fracture resistance values (2770 ± 598 N) of 0.6 mm-lithium disilicate occlusal veneers bonded to dentin. Additionally, Huang et al. [14] reported similar fracture load for lithium disilicate occlusal veneers, and Zhang et al. [11] reported a fracture load of 2249 ± 375 N for lithium disilicate occlusal veneers. However, another study reported lower values for lithium disilicate and polymer-infiltrated ceramic occlusal veneers which may be related to tooth type and testing of occlusal veneers with a fissure/cusp thickness of 0.5/0.8 mm [22].

In the present study, occlusal veneer with intra-coronal cavity preparation design was studied. Occlusal cavity preparation is needed when a tooth develops carious lesions, fractures, or loses a large tooth substance because of abrasion or erosion as the suitable prepared cavities allow for a restorative material placement [8,13]. Adhesively bonded filings are needed to compensate deep defects, strengthen tooth structure, enable good adhesive bond with dentin, decrease the cuspal flexure, and assure a uniform ceramic thickness over the filling [3,6,8,13,17]. High occlusal forces, on the other hand, can cause stress concentration at the filler-resin matrix interface, resulting in filler dislodgment and resin matrix exposure, resulting in wear that would necessitate a prosthetic procedure [6,57].

Regarding the influence of the dental bonding substrates, there was no significant difference between the tested substrates. A study found that occlusal veneers cemented to enamel or dentin had fracturing resistance that was equal to that of a sound tooth [41]. A study investigated the fracture resistance of lithium disilicate occlusal veneer restorations bonded to various dental bonding surfaces, finding no substantial differences in fracture resistance between lithium disilicate occlusal veneer restorations (0.7–1 mm thickness) bonded to enamel and dentin, or enamel and dentin with composite filling [38]. Another study demonstrated that marginal sealing of ceramic inlays bonded to composite is comparable to bonding to dentin when the composite filling is present as a bonding substrate [58].

The current research found no statistically meaningful differences between the LC, ZC, and PC groups. The fracture resistance value in the LC group was the highest. The fracture resistance of lithium disilicate occlusal veneers with an intra-coronal cavity is investigated in a published study [16]. They found that occlusal veneers with 1 mm cuspal thickness and 1.7 mm fissure depth as an intra-coronal cavity formed part of the restoration have higher (but not significant) fracture resistance than that of the restorations with 1 mm cuspal thickness and 0.7 mm fissure depth where a composite filling was used to restore the intra-coronal cavity. This supports the assumption that thicker ceramics increases the strength and creating a clinically resilient restoration [19]. Additionally, increasing the zirconia thickness enhanced its resistance to fracture [24,25].

The most common failure pattern in the PD and LD groups was catastrophic failure, which included both the restoration and the tooth, suggesting that these restorations are at least as strong as the worn teeth they are supposed to restore [15]. A comparable elastic modulus between the substrate and the restorative material favors the tooth-restoration complex [40]. Specimens with polymer-infiltrated ceramics exhibited greater tooth structure deformation which could be related to the load which exceeded the elastic limit of the restoration and tooth [28]. It was reported that failure including both ceramics and tooth with lithium disilicate occlusal veneers bonded to dentin as the predominant failure mode [39]. Additionally, the reliable bonding with tooth structure for either glass-containing ceramics or polymer-infiltrated ceramics enables force transmission through the restoration-tooth complex [5,35]. The applied forces, on the other hand, may cause the crack formation and spreading, resulting in fracturing and structural collapse that may spread to the tooth structure [7]. Huang et al. [59] concluded that the stress within lithium disilicate occlusal veneer was 1.5 times higher than for polymer-infiltrated ceramics occlusal veneer.

Regarding the PF group, less complicated fractures were observed, and the presence of composite filling underneath the restoration may contribute to the longevity of the underlying tooth [16]. Furthermore, structures with compatible elastic moduli bend less under load and allocate stresses more equally, avoiding damage to the underlying tooth structure [28]. However, the class IV failure mode was dominant with the presence of intra-coronal cavity. This could be related to sacrificing in tooth structure causing a reduction in the strength of the remaining tooth structure [16,39].

Zirconia restorations, on the other hand, had a lower rate of catastrophic failure, which may be due to the difficulty of bonding it to tooth structure [25]. Additionally, zirconia groups are the only ones which experienced a class III failure pattern. It was reported that de-bonding was the most common long-term failure observed for zirconia restoration [23]. Mendes et al. [60] evaluated the stresses in occlusal veneer according to the restoration type and found higher stress concentration in zirconia followed by lithium disilicate and polymer-infiltrated ceramics, and also they found that the stress on the cement layer was greatest with polymer-infiltrated ceramics followed by zirconia and lithium disilicate.

Since there are more thickness differences in clinical use, the current study only focused at one thickness of non-retentive occlusal veneers. Variable thicknesses need to be tested. Other designs, as well as diverse types of resin cements, must be tested in relation to various dental bonding surfaces. In the present research, the teeth were prepared. Sclerotic dentin covers the occlusal surfaces of worn teeth, and the adhesive bonding can vary. Furthermore, clinical studies are expected to predict occlusal veneers’ clinical results.

## 5. Conclusions

Despite the limitations of this study, the following conclusions could be obtained:

1. Considering the fracture results, lithium disilicate, zirconia, and polymer-infiltrated ceramic occlusal veneers perform well whatever the type of dental bonding surface.
2. When the dental bonding surface varies, different occlusal veneer materials should be considered.
3. Occlusal veneers bonded to dentin, dentin with composite filling, or dentin with an intra-coronal cavity exhibited a fracture resistance exceeding the average human masticatory forces in the molar area. Therefore, these occlusal veneers might be a tooth substance preserving option for restoring the occlusal surfaces of posterior teeth.

## Figures and Tables

**Table 1:** Materials used in the study.

Material	Product Name	Lot No.	Main Composition	Manufacturer
**Lithium disilicate**	IPS e.max CAD	S25999	SiO_2_ (57–80%), Li_2_O (11–19%), K_2_O (0–13%), P_2_O_5_ (0–11%), ZrO_2_ (0–8%), ZnO (0–8%) and coloring oxides (0–12%)	Ivoclar Vivadent, Schaan, Liechtenstein
**Translucent zirconia**	Katana UTML Zirconia	228001002	ZrO_2_ and HfO_2_ (86.3–94.2%), Er_2_O_3_ (>2%), Fe_2_O_3_ (6.07%), Y_2_O_3_ (5.8–9.7%), Al_2_O_3_ (<0.5%)	Kuraray Noritake Dental Inc., Tokyo, Japan
**Polymer-infiltrated ceramic**	Vita Enamic	82960	Ceramic part: Silicon dioxide (58–63%), Aluminum oxide (20–23%), Sodium oxide (9–11%), Potassium oxide (4–6%), Boron trioxide (0.5–2%), Zirconia (<1%), Calcium oxide (Calcium oxide (<1%). Polymer part: urethane dimethacrylate, triethylene glycol dimethacrylate.	VITA Zahnfabrik, Bad Säckingen, Germany
**Bonding agent**	All-Bond Universal	2000001141	Bisphenol A Diglycidylmethacrylate (20–50%), Ethanol (30–50%), Methacryloyloxydecyl dihydrogen phosphate (5–25%), 2-Hydroxyethyl Methacrylate (2–25%), water, Initiators	Bisco Inc. Schaumburg, IL, USA
**Ceramic primer**	Porcelain Primer	2000000727	3-(Trimethoxysilyl)propyl-2-Methyl-2-PropenoicAcid (1–5%), Ethanol (30–50%), Acetone (30–50%)	Bisco Inc. Schaumburg, IL, USA
**Zirconia primer**	Z-Prime Plus	200000745	10-Methacryloyloxydecyl Dihydrogen Phosphate (1–5%), 2-Hydroxyethyl Methacrylate (5–10%), Bisphenol A, Ethanol (75–85%)	Bisco Inc. Schaumburg, IL, USA
**Resin cement**	Duo-Link Universal	2000001243	Base: Bisphenol A Diglycidylmethacrylate (10–30%), Urethane Dimethacrylate (10–30%), Ytterbium Oxide-Silica (1–5%), Tetrahydrofurfuryl Methacrylate (1–5%), Trimethylolpropane Trimethacrylate (1–5%), Ytterbium Fluoride (10–20%), Catalyst: Bisphenol A Diglycidylmethacrylate (10–30%), Dibenzoyl Peroxide (<1%)	Bisco Inc. Schaumburg, IL, USA

**Table 2:** Study grouping.

Code	Group	
	Material Type	Bonding Surfaces
**LD**	Lithium disilicate	Dentin
**LC**	Lithium disilicate	Dentin with intra-coronal cavity
**LF**	Lithium disilicate	Dentin with composite filling
**ZD**	Zirconia	Dentin
**ZC**	Zirconia	Dentin with intra-coronal cavity
**ZF**	Zirconia	Dentin with composite filling
**PD**	Polymer-infiltrated ceramic	Dentin
**PC**	Polymer-infiltrated ceramic	Dentin with intra-coronal cavity
**PF**	Polymer-infiltrated ceramic	Dentin with composite filling

**Table 3:** Mean and standard deviation of fracture resistance values (N) of the studied groups.

	Lithium Disilicate	Zirconia	Polymer-Infiltrated Ceramic	Sig.
Bonded to dentin	2251.05 ± 604.89	2285.18 ± 491.06	2505.62 ± 600.89	0.560
Bonded to dentin with intra-coronal cavity	2505.91 ± 723.01	2073.32 ± 426.45	1947.13 ± 650.45	0.121
Bonded to dentin with composite filling	2182.53 ± 643.77	1859.88 ± 422.75	2364.55 ± 648.14	0.164
Sig.	0.521	0.124	0.142	-

**Table 4:** Summary of two-way ANOVA test representing the interaction between the study variables.

Source	Type III Sum of Squares	df	Mean Square	F	Sig.
Corrected Model	4.165 ^a^	8	520,620.129	1.507	0.168
Intercept	4.433	1	4.433	1283.38	<0.001
Veneer material	992,911.013	2	496,455.506	1.437	0.244
Bonding surface	758,971.882	2	379,485.941	1.099	0.338
Veneer material X Bonding surface	2,413,078.137	4	603,269.534	1.746	0.148
Error	2.798	81	345,447.907	-	-
Total	4.755	90	-	-	-
Corrected Total	3.215	89	-	-	-

^a^: R Squared = 0.130 (Adjusted R Squared = 0.044).

**Table 5:** Failure mode percentage (%) among study groups.

Failure Mode		Lithium Disilicate		Zirconia		Polymer-Infiltrated Ceramic		Sig.
		N	%	N	%	N	%	
Bonded to dentin	Class I	0	0.0	0	0.0	3	30.0	0.004
	Class II	3	30.0	3	30.0	0	0.0	
	Class III	0	0	4	40.0	0	0.0	
	Class IV	7	70.0	3	30.0	7	70.0	
Bonded to dentin with intra-coronal cavity	Class I	2	20.0	0	0.0	1	10.0	0.068
	Class II	2	20.0	2	20.0	1	10.0	
	Class III	0	0.0	4	40.0	0	0.0	
	Class IV	6	60.0	4	40.0	8	80.0	
Bonded to dentin with composite filling	Class I	2	20.0	0	0.0	4	40	0.012
	Class II	3	30.0	0	0.0	0	0.0	
	Class III	0	0.0	3	30.0	0	0.0	
	Class IV	5	50.0	7	70.0	6	60.0	
Sig.		0.630		0.304		0.401		-

## Data Availability

The data presented in this study are available on request from the corresponding author W.A.-Z.

## References

[1] Resende T.H., Reis K.R., Schlichting L.H., Magne P. (2018). Ultrathin CAD-CAM Ceramic Occlusal Veneers and Anterior BilaminVeneers for the Treatment of Moderate Dental Biocorrosion: A 1.5-Year Follow-Up. Oper. Dent..

[2] Muts E.J., van Pelt H., Edelhoff D., Krejci I., Cune M. (2014). Tooth wear: A systematic review of treatment options. J. Prosthet. Dent..

[3] Freitas A.C., Silva A.M., Lima Verde M.A., Jorge de Aguiar J.R. (2012). Oral rehabilitation of severely worn dentition using an overlay for immediate re-establishment of occlusal vertical dimension. Gerodontology.

[4] Vailati F., Belser U.C. (2008). Full-mouth adhesive rehabilitation of a severely eroded dentition: The three-step technique. Part 1. Eur. J. Esthet. Dent..

[5] Cardoso M.V., de Almeida Neves A., Mine A., Coutinho E., Van Landuyt K., De Munck J., Van Meerbeek B. (2011). Current aspects on bonding effectiveness and stability in adhesive dentistry. Aust. Dent. J..

[6] Fradeani M., Barducci G., Bacherini L., Brennan M. (2012). Esthetic rehabilitation of a severely worn dentition with minimally invasive prosthetic procedures (MIPP). Int. J. Periodontics Restor. Dent..

[7] Kwon S.J., Lawson N.C., McLaren E.E., Nejat A.H., Burgess J.O. (2018). Comparison of the mechanical properties of translucent zirconia and lithium disilicate. J. Prosthet. Dent..

[8] Rocca G.T., Rizcalla N., Krejci I., Dietschi D. (2015). Evidence-based concepts and procedures for bonded inlays and onlays. Part II. Guidelines for cavity preparation and restoration fabrication. Int. J. Esthet. Dent..

[9] Walter R., Swift E.J., Boushell L.W., Braswell K. (2011). Enamel and dentin bond strengths of a new self-etch adhesive system. J. Esthet. Restor. Dent..

[10] Maeder M., Pasic P., Ender A., Özcan M., Benic G.I., Ioannidis A. (2019). Load-bearing capacities of ultra-thin occlusal veneers bonded to dentin. J. Mech. Behav. Biomed. Mater..

[11] Zhang H., Lv P., Du W., Jiang T. (2020). Comparison of Fracture Load and Surface Wear of Microhybrid Composite and Ceramic Occlusal Veneers. J. Prosthodont..

[12] Lussi A., Hellwig E., Ganss C., Jaeggi T. (2009). Buonocore Memorial Lecture. Dental erosion. Oper. Dent..

[13] Ahlers M.O., Mörig G., Blunck U., Hajtó J., Pröbster L., Frankenberger R. (2009). Guidelines for the preparation of CAD/CAM ceramic inlays and partial crowns. Int. J. Comput. Dent..

[14] Huang X., Zou L., Yao R., Wu S., Li Y. (2020). Effect of preparation design on the fracture behavior of ceramic occlusal veneers in maxillary premolars. J. Dent..

[15] Johnson A.C., Versluis A., Tantbirojn D., Ahuja S. (2014). Fracture strength of CAD/CAM composite and composite-ceramic occlusal veneers. J. Prosthodont. Res..

[16] Lierop J., Moodley D., Mulder R. (2019). Influence of ceramic thickness and cavity design optimization on fracture resistance of partial coverage restorations. N. Z. Dent. J..

[17] Veneziani M. (2017). Posterior indirect adhesive  restorations: Updated indications  and the Morphology Driven  Preparation Technique. Int. J. Esthet. Dent..

[18] Al-Akhali M., Kern M., Elsayed A., Samran A., Chaar M.S. (2019). Influence of thermomechanical fatigue on the fracture strength of CAD-CAM-fabricated occlusal veneers. J. Prosthet. Dent..

[19] Bajraktarova-Valjakova E., Korunoska-Stevkovska V., Kapusevska B., Gigovski N., Bajraktarova-Misevska C., Grozdanov A. (2018). Contemporary Dental Ceramic Materials, A Review: Chemical Composition, Physical and Mechanical Properties, Indications for Use. Open Access Maced. J. Med. Sci..

[20] Edelhoff D., Sorensen J.A. (2002). Tooth structure removal associated with various preparation designs for posterior teeth. Int. J. Periodontics Restor. Dent..

[21] Bidra A.S., Pieger S., Salman A., Bidra A.S. (2014). Clinical outcomes of lithium disilicate single crowns and partial fixed dental prostheses: A systematic review. J. Prosthet. Dent.

[22] Al-Akhali M., Chaar M.S., Elsayed A., Samran A., Kern M. (2017). Fracture resistance of ceramic and polymer-based occlusal veneer restorations. J. Mech. Behav. Biomed. Mater..

[23] Miyazaki T., Nakamura T., Matsumura H., Ban S., Kobayashi T. (2013). Current status of zirconia restoration. J. Prosthodont. Res..

[24] Nakamura K., Harada A., Inagaki R., Kanno T., Niwano Y., Milleding P., Örtengren U. (2015). Fracture resistance of monolithic zirconia molar crowns with reduced thickness. Acta. Odontol. Scand..

[25] Weigl P., Sander A., Wu Y., Felber R., Lauer H.C., Rosentritt M. (2018). In-vitro performance and fracture strength of thin monolithic zirconia crowns. J. Adv. Prosthodont..

[26] Sakrana A.A., Al-Zordk W., El-Sebaey H., Elsherbini A., Özcan M. (2021). Does Preheating Resin Cements Affect Fracture Resistance of Lithium Disilicate and Zirconia Restorations?. Materials.

[27] Al-Zordk W., Saker S. (2020). Impact of sintering procedure and clinical adjustment on color stability and translucency of translucent zirconia. J. Prosthet. Dent..

[28] Coldea A., Swain M.V., Thiel N. (2013). Mechanical properties of polymer-infiltrated-ceramic-network materials. Dent. Mater..

[29] Facenda J.C., Borba M., Corazza P.H. (2018). A literature review on the new polymer-infiltrated ceramic-network material (PICN). J. Esthet. Restor. Dent..

[30] Magne P., Schlichting L.H., Maia H.P., Baratieri L.N. (2010). In vitro fatigue resistance of CAD/CAM composite resin and ceramic posterior occlusal veneers. J. Prosthet. Dent..

[31] Magne P. (2014). IDS: Immediate Dentin Sealing (IDS) for tooth preparations. J. Adhes. Dent..

[32] Angerame D., De Biasi M., Agostinetto M., Franzò A., Marchesi G. (2019). Influence of preparation designs on marginal adaptation and failure load of full-coverage occlusal veneers after thermomechanical aging simulation. J. Esthet. Restor. Dent..

[33] Federlin M., Krifka S., Herpich M., Hiller K.A., Schmalz G. (2007). Partial ceramic crowns: Influence of ceramic thickness, preparation design and luting material on fracture resistance and marginal integrity in vitro. Oper. Dent..

[34] Clausen J.O., Abou Tara M., Kern M. (2010). Dynamic fatigue and fracture resistance of non-retentive all-ceramic full-coverage molar restorations. Influence of ceramic material and preparation design. Dent. Mater..

[35] Qanungo A., Aras M.A., Chitre V., Mysore A., Amin B., Daswani S.R. (2016). Immediate dentin sealing for indirect bonded restorations. J. Prosthodont. Res..

[36] Yazigi C., Kern M., Chaar M.S. (2017). Influence of various bonding techniques on the fracture strength of thin CAD/CAM-fabricated occlusal glass-ceramic veneers. J. Mech. Behav. Biomed. Mater..

[37] Malament K.A., Socransky S.S. (2001). Survival of Dicor glass-ceramic dental restorations over 16 years. Part III: Effect of luting agent and tooth or tooth-substitute core structure. J. Prosthet. Dent..

[38] Sasse M., Krummel A., Klosa K., Kern M. (2015). Influence of restoration thickness and dental bonding surface on the fracture resistance of full-coverage occlusal veneers made from lithium disilicate ceramic. Dent. Mater..

[39] Krummel A., Garling A., Sasse M., Kern M. (2019). Influence of bonding surface and bonding methods on the fracture resistance and survival rate of full-coverage occlusal veneers made from lithium disilicate ceramic after cyclic loading. Dent. Mater..

[40] Heck K., Paterno H., Lederer A., Litzenburger F., Hickel R., Kunzelmann K.H. (2019). Fatigue resistance of ultrathin CAD/CAM ceramic and nanoceramic composite occlusal veneers. Dent. Mater..

[41] Valenzuela E.B.S., Andrade J.P., da Cunha P.F.J.S., Bittencourt H.R., Spohr A.M. (2021). Fracture load of CAD/CAM ultrathin occlusal veneers luted to enamel or dentin. J. Esthet. Restor. Dent..

[42] Andrade J.P., Stona D., Bittencourt H.R., Borges G.A., Burnett L.H., Spohr A.M. (2018). Effect of Different Computer-aided Design/Computer-aided Manufacturing (CAD/CAM) Materials and Thicknesses on the Fracture Resistance of Occlusal Veneers. Oper. Dent..

[43] Baldissara P., Monaco C., Onofri E., Fonseca R.G., Ciocca L. (2019). Fatigue resistance of monolithic lithium disilicate occlusal veneers: A pilot study. Odontology.

[44] Chen C., Trindade F.Z., de Jager N., Kleverlaan C.J., Feilzer A.J. (2014). The fracture resistance of a CAD/CAM Resin Nano Ceramic (RNC) and a CAD ceramic at different thicknesses. Dent. Mater..

[45] Chen S.E., Park A.C., Wang J., Knoernschild K.L., Campbell S., Yang B. (2019). Fracture Resistance of Various Thickness e.max CAD Lithium Disilicate Crowns Cemented on Different Supporting Substrates: An In Vitro Study. J. Prosthodont..

[46] Ioannidis A., Mühlemann S., Özcan M., Hüsler J., Hämmerle C.H.F., Benic G.I. (2019). Ultra-thin occlusal veneers bonded to enamel and made of ceramic or hybrid materials exhibit load-bearing capacities not different from conventional restorations. J. Mech. Behav. Biomed. Mater..

[47] Yazigi C., Schneider H., Chaar M.S., Rüger C., Haak R., Kern M. (2018). Effects of artificial aging and progression of cracks on thin occlusal veneers using SD-OCT. J. Mech. Behav. Biomed. Mater..

[48] Morresi A.L., D’Amario M., Capogreco M., Gatto R., Marzo G., D’Arcangelo C., Monaco A. (2014). Thermal cycling for restorative materials: Does a standardized protocol exist in laboratory testing? A literature review. J. Mech. Behav. Biomed. Mater..

[49] Nawafleh N., Hatamleh M., Elshiyab S., Mack F. (2016). Lithium Disilicate Restorations Fatigue Testing Parameters: A Systematic Review. J. Prosthodont..

[50] Guess P.C., Schultheis S., Wolkewitz M., Zhang Y., Strub J.R. (2013). Influence of preparation design and ceramic thicknesses on fracture resistance and failure modes of premolar partial coverage restorations. J. Prosthet. Dent..

[51] Comba A., Baldi A., Tempesta R.M., Carossa M., Perrone L., Saratti C.M., Rocca G.T., Femiano R., Femiano F., Scotti N. (2021). Do Chemical-Based Bonding Techniques Affect the Bond Strength Stability to Cubic Zirconia?. Materials.

[52] Salem R.S.T., Ozkurt-Kayahan Z., Kazazoglu E. (2019). In Vitro Evaluation of Shear Bond Strength of Three Primer/Resin Cement Systems to Monolithic Zirconia. Int. J. Prosthodont..

[53] Yagawa S., Komine F., Fushiki R., Kubochi K., Kimura F., Matsumura H. (2018). Effect of priming agents on shear bond strengths of resin-based luting agents to a translucent zirconia material. J. Prosthodont. Res..

[54] Gibbs C.H., Mahan P.E., Mauderli A., Lundeen H.C., Walsh E.K. (1986). Limits of human bite strength. J. Prosthet. Dent..

[55] Waltimo A., Nyström M., Könönen M. (1994). Bite force and dentofacial morphology in men with severe dental attrition. Scand. J. Dent. Res..

[56] Ferrario V.F., Sforza C., Serrao G., Dellavia C., Tartaglia G.M. (2004). Single tooth bite forces in healthy young adults. J. Oral Rehabil..

[57] Rosatto C.M., Bicalho A.A., Veríssimo C., Bragança G.F., Rodrigues M.P., Tantbirojn D., Versluis A., Soares C.J. (2015). Mechanical properties, shrinkage stress, cuspal strain and fracture resistance of molars restored with bulk-fill composites and incremental filling technique. J. Dent..

[58] Müller V., Friedl K.H., Friedl K., Hahnel S., Handel G., Lang R. (2017). Influence of proximal box elevation technique on marginal integrity of adhesively luted Cerec inlays. Clin. Oral Investig..

[59] Huang X.Q., Hong N.R., Zou L.Y., Wu S.Y., Li Y. (2020). Estimation of stress distribution and risk of failure for maxillary premolar restored by occlusal veneer with different CAD/CAM materials and preparation designs. Clin. Oral Investig..

[60] Mendes J.P., de Oliveira A.M., Moreira M., Souto A.L., Antonio M. (2018). Influence of ceramic material, thickness of restoration and cement layer on stress distribution of occlusal veneers. Braz. Oral Res..

